# Editorial: Enhancing plant stress resilience: innovative roles of plant growth-promoting bacteria (PGPB) in sustainable agriculture and crop protection

**DOI:** 10.3389/fpls.2026.2023684

**Published:** 2026-10-27

**Authors:** Carlos Henrique S. G. Meneses, Jean Luiz Simoes-Araujo, Krzysztof Szczyglowski, Marcia Soares Vidal

**Affiliations:** 1 Laboratory Plant Biotechnology, Graduate Program in Agricultural Sciences, Department of Biology, Center for Biological and Health Sciences, State University of Paraíba, Campina Grande, Brazil; 2 Laboratory of Genetics and Biochemistry, Empresa Brasileira de Pesquisa Agropecuária – EMBRAPA, Embrapa Agrobiologia, Seropédica, Rio de Janeiro, Brazil; 3 London Research and Development Centre, Agriculture and Agri-Food Canada (AAFC), London, ONT, Canada

**Keywords:** biological control, biotic and abiotic stress mitigation, microbial inoculants, plant-microbe synergy, rhizosphere microbiome

Given significant global climate change, enhancing plant resilience to multiple stresses (both abiotic and biotic) is essential for sustainable agriculture. Plants face challenges related to nutrients, pathogens, drought, and extreme temperatures, factors that threaten their growth and productivity. Traditional agricultural practices, when applied in isolation, are often insufficient to withstand these stresses. Consequently, innovative biological solutions, such as the use of beneficial microorganisms, are crucial for increasing plant growth and tolerance to stress.

Elucidating the dynamics of plant-microorganism interactions under diverse environmental conditions remains a challenging yet essential task for unlocking the full potential of microorganisms in agriculture. However, despite existing findings, many questions regarding the molecular crosstalk in plant-microbe interactions remain unanswered, with intriguing discoveries awaiting further insight.

With a shared commitment to improving crop performance and advancing sustainable agriculture, we present a Research Topic that explore plant-microbe interactions and rhizosphere processes. These contributions expand our understanding of microbial diversity and its potential to improve plant nutrition, alleviate environmental stresses, support biological control, and reduce the environmental impacts of agricultural production. This Research Topic brings together 12 original articles focusing on: (i) the prospecting and characterization of plant growth-promoting bacteria, with an emphasis on mechanisms for mitigating water deficit and on the evaluation of inoculants under controlled and field conditions; (ii) the use of bacterial strains and consortia to improve nutrient acquisition, fertilizer use efficiency, and crop productivity; (iii) the investigation of the taxonomic, functional, and genomic diversity of beneficial bacteria, including the evolutionary analysis of genes; (iv) the application of bacteria for the biological control of phytopathogens and root-knot nematodes, including mechanisms interfering with bacterial communication; (v) the contribution of endophytic fungi to plant growth and cadmium tolerance through alterations in root architecture and the chemical forms of the metal; and (vi) understanding the relationships between soil properties, microbial communities, and rhizosphere interactions, covering plant responses to drought and strategies for reducing nitrous oxide emissions using nitrification inhibitors.

Regarding the first topic presented, Vasconcelos et al. report that inoculating soybean seeds with *Bacillus velezensis* strains 5D5 and 6E9 and *Bacillus subtilis* strain 1A11 promotes biomass accumulation and increases grain yield by 11.3% to 18% under water deficit conditions, with monitoring validated via remote sensing using the EVI2 index. Meanwhile, Ferreira et al. demonstrate that ACC deaminase-producing bacteria (*Paraburkholderia silvatlantica* strain NRB142, *Azospirillum melinis* strain NRB223, and *Herbaspirillum frisingense* strain BR11790) reduce the impacts of water stress and stimulate plant development in *Brachiaria*. Similarly, Zhang et al. isolated rhizospheric bacteria from winter wheat (*Microbacterium* sp. I2, *Arthrobacter* sp. R4, and *Microbacterium* sp. K2) and identified genetic and physiological mechanisms, such as the trehalose pathway, IAA synthesis, N fixation, and P solubilization, that support plant growth under drought conditions. Finally, Ishaq et al. characterized rhizobacteria isolated from the Uzungöl forest [such as *Herbaspirillum huttiense* strain S1(E) and *Pseudomonas mohnii* strain SS7(5)], showing that seed biopriming and root inoculation stimulate germination vigor and early growth of rice in a hydroponic system. In the context of adverse climate scenarios and water scarcity, these studies offer crucial biological alternatives to ensure agricultural productivity.

Considering the second topic, Beltrán-Medina et al. show that the inoculation of basil with PGPB consortia (such as *Herbaspirillum* sp. AP21 + *Azospirillum brasilense* strain D7 + *Rhizobium leguminosarum* strain T88, or *Rhizobium* sp. B02 + *Azotobacter chroococcum* strains AC1 + AC10) makes it possible to reduce N and P mineral fertilization by 50%, maintaining or overcoming biomass accumulation and absorption of nutrients observed in complete conventional fertilization. On the other hand, Bernal-Vicente et al. describe the characterization of the new endophytic strain *Pseudomonas seleniipraecipitans* strain ABP-B9, demonstrating its biostimulant action on lettuce, spinach and celery by optimizing the development of the root system, photosynthetic efficiency, flavonoid contents and commercial productivity under field management. Taken together, this research highlights the potential of beneficial microorganisms to optimize the nutritional use of crops and promote low chemical input agriculture.

Within the scope of the third topic, Terra et al. conducted a pangenomic analysis of 44 symbiotic genomes from the genus *Bradyrhizobium*, revealing an open pangenome with extensive genomic plasticity, a probable evolutionary origin in the Northern Hemisphere, and strong biogeographical and phylogenetic structuring associated with genes involved in nodulation, biological nitrogen fixation, and the type III secretion system. These studies expand our understanding of the evolution of legume symbiosis and the functional diversity of bacteria adapted to environmental stresses.

Observing the fourth topic, Sánchez et al. performed a polyphasic and taxagenomic characterization of the new species *Pseudomonas halotolerans* sp. nov. strain B22^T^, isolated from the rhizosphere of the halophyte *Salicornia hispanica*, highlighting its plant-growth-promoting properties in tomato plants through the production of IAA and siderophores, as well as phosphate solubilization and effectively controls bacterial phytopathogens (such as *Dickeya solani*, *Pectobacterium* spp., *Pantoea agglomerans*, and *Pseudomonas syringae*) by degrading AHL molecules via Quorum Quenching, thereby attenuating the severity of soft rot in vegetables and fruits without inhibiting pathogen cell growth. Concurrently, Mata et al. developed a bacterial consortium composed of *Bacillus amyloliquefaciens* UC_2.4, *Pseudomonas capeferrum* UC_21.3 A.1, and *Pseudomonas capeferrum* UC_21.30 A.1; this consortium demonstrated 100% *in vitro* nematicidal efficacy against *Meloidogyne hapla* and reduced infection in tomato plants threefold, while maintaining highly selective action that preserved the beneficial, non-target, free-living nematode *Caenorhabditis elegans*. Both approaches represent sustainable, targeted biotechnological strategies for replacing synthetic pesticides in pest and disease management.

In the fifth topic presented, Lin et al. investigated the contribution of the dark septate endophyte *Paraboeremia selaginellae* to the growth of the medicinal plant *Salvia miltiorrhiza* in cadmium-contaminated soil, demonstrating that the fungus stimulates root architecture development (length, area, and volume) and increases the activity of soil enzymes such as sucrase, urease, and alkaline phosphatase. Additionally, the authors demonstrated that inoculation reduces metal phytotoxicity by altering the distribution of cadmium chemical forms in the soil, thereby decreasing its harmful uptake by the plant. This research highlights the strategic role of endophytic fungi in the phytostabilization and ecological recovery of agricultural ecosystems and heavy metal-polluted soils.

Finally, in the sixth Research Topic, Zhang et al. evaluated how soil properties and rice developmental stages affect rhizospheric nitrous oxide emissions, demonstrating that the use of nitrification inhibitors significantly reduces nitrification rates and nitrous oxide accumulation by altering the relative abundance of nitrifying microbial communities. Meanwhile, Wang et al. analyzed the responses of the *Salvia miltiorrhiza* rhizosphere microbiota to drought stress, observing that the plant reorganizes its microbial co-occurrence network around hub nodes dominated by tolerant bacteria (such as *Alcaligenes faecalis* and *Bacillus pseudomycoides*) and recruits functions related to N fixation, P solubilization, and K release. These findings shed light on the complex interactions among soil, microorganisms, and roots, providing insights for enhancing agricultural resilience and mitigating greenhouse gas emissions.

The articles in this Research Topic expand our understanding of plant-microorganism interactions, highlighting the microbiota potential to improve plant nutrition, mitigate the effects of drought and cadmium toxicity, and facilitate biological control. Genomic analyses and rhizosphere communities studies contribute to understanding symbiotic associations and guiding strategies to reduce nitrous oxide emissions. Validating proposed mechanisms and broadening evaluations across different crops and environments will help consolidate these advances, supporting the development of bio-inputs and management practices that balance productivity, crop protection, and environmental conservation.

